# Complications and in-hospital mortality in trauma patients treated in intensive care units in the United States, 2013

**DOI:** 10.1186/s40621-016-0084-5

**Published:** 2016-08-04

**Authors:** Meghan Prin, Guohua Li

**Affiliations:** 1 Department of Anesthesiology & Critical Care, Columbia University College of Physicians and Surgeons, 622 West 168th Street, PH 505, New York, NY 10032 USA; 2 Department of Epidemiology, Columbia University Mailman School of Public Health, New York, NY USA

**Keywords:** Trauma, Hospitalization, Intensive care unit, Critical care, Complications

## Abstract

**Background:**

Traumatic injury is a leading cause of morbidity and mortality worldwide, but epidemiologic data about trauma patients who require intensive care unit (ICU) admission are scant. This study aimed to describe the annual incidence of ICU admission for adult trauma patients, including an assessment of risk factors for hospital complications and mortality in this population.

**Methods:**

This was a retrospective study of adults hospitalized at Level 1 and Level 2 trauma centers after trauma and recorded in the National Trauma Data Bank in 2013. Multiple logistic regression analyses were performed to determine predictors of hospital complications and hospital mortality for those who required ICU admission.

**Results:**

There were an estimated total of 1.03 million ICU admissions for trauma at Level 1 and Level 2 trauma centers in the United States in 2013, yielding an annual incidence of 3.3 per 1000 population. The annual incidence was highest in men (4.6 versus 1.9 per 100,000 for women), those aged 80 years or older (7.8 versus 3.6–4.3 per 100,000 in other age groups), and residents in the Western US Census region (3.9 versus 2.7 to 3.6 per 100,000 in other regions). The most common complications in patients admitted to the ICU were pneumonia (10.9 %), urinary tract infection (4.7 %), and acute respiratory distress syndrome (4.4 %). Hospital mortality was significantly higher for ICU patients who developed one or more complications (16.9 % versus 10.7 % for those who did not develop any complications, *p* < 0.001).

**Conclusions:**

Admission to the ICU after traumatic injury is common, and almost a quarter of these patients experience hospital complications. Hospital complications are associated with significantly increased risk of mortality.

**Electronic supplementary material:**

The online version of this article (doi:10.1186/s40621-016-0084-5) contains supplementary material, which is available to authorized users.

## Background

Trauma is a major cause of morbidity and mortality in the United States. In 2013 it was the leading cause of death for people ages 35–44, and the fourth leading cause of death for the whole population (Murphy et al. 2016). Trauma includes intentional and unintentional injury from motor vehicle crashes, penetrating or blunt violence, falls, firearms, poisoning, and burns. Pre-hospital systems have been studied extensively to optimize the initial care of trauma patients (Williams et al. 2013; McQueen et al. 2015; McNeill and Bryden 2013; Ringburg et al. 2009; Wilson et al. 2015; National Institute for Health and Care Excellence 2016), but there is a shortage of data describing the clinical course of patients admitted to the intensive care unit (ICU) after traumatic injury. The aim of this study was to describe the clinical characteristics of adult patients admitted to the ICU after traumatic injury, including an assessment of the risk factors for hospital complications and hospital mortality in this population.

## Methods

This was a population-based, multicenter retrospective study of adult patients admitted to the ICU at Level 1 and Level 2 trauma centers after traumatic injury in 2013, using the National Trauma Data Bank (NTDB) National Sample Program. The NTDB is the largest registry of trauma data in the United States, and the National Sample Program includes data from a nationally representative sample of 100 hospitals with Level 1 or Level 2 American College of Surgery (ACS) Trauma designation. The NTDB National Sample Program data are collected prospectively by trained data abstractors at contributing hospitals, and validated centrally before pooling. Steps taken to ensure the validity of the data include the use of a data dictionary, data collection tutorials, and electronic validation of incomplete or inconsistent data. Inclusion and exclusion criteria vary between contributing hospitals. All patients were followed until hospital discharge. The National Sample Program uses a stratified sampling design, with 16 strata based on United States Census regions (Northeast, Midwest, South, and West), level of trauma care designation (ACS Levels 1 and 2), and NTDB reporting status (NTDB-contributors, NTDB-non-contributors). The final weights for hospitals are also adjusted by Emergency Room monthly volume.

Data collected as part of the National Sample Program include general patient demographics, pre-hospital emergency medical services, trauma severity scores (Injury Severity Score (ISS) (Baker et al. 1974)), injury class (e.g., blunt versus penetrating), intention (assault, self-harm, unintentional, other), Emergency Room vital signs (e.g., blood pressure, Glasgow Coma Scale Score (GCS) (Teasdale and Jennett 1974)), preexisting clinical diagnoses, in-hospital diagnosis codes, hospital length of stay, intensive care unit length of stay, and discharge status (mortality, discharge destination). Patients were included for analysis if they were 18 years or older and were admitted to the hospital. Patients were excluded if they were discharged from or died in the Emergency Room. Patients with primary burn injuries were also excluded, because Injury Severity Scores are not validated in this population and major burn injuries are frequently cared for at designated burn centers rather than trauma centers (Pruitt et al. 2012).

We first assessed transitions of care by summarizing the locations of patients after hospital admission from the Emergency Room. We were unable to assess the temporal flow of patient transitions during the hospitalization. We were also unable to account for or exclude readmissions, and patients may have been admitted more than once during the sampling period. We then summarized the national incidence rates of ICU admission with 95 % confidence intervals (CI) based on United States 2013 population census data by age, gender, race, injury type, and census region (Appendix, Table 5) (United States Census Bureau 2013).

We divided all admissions into two groups: patients admitted to the ICU during the hospital course and patients not admitted to the ICU during the hospital course. We defined those who were admitted to the ICU as patients who were admitted to ICU at any time during the index hospitalization, not necessarily those admitted directly to the ICU from the Emergency Room. We summarized the general demographics (e.g., age, gender), clinical scores (e.g., Glasgow Coma Scale, Injury Severity Score) injury types (e.g., blunt, penetrating, other) and the presence of hypotension on admission (systolic blood pressure < 90 mmHg), which has been demonstrated as a prognostic factor in trauma patients (Parks et al. 2006). We summarized the proportion of patients who received mechanical ventilation. We assessed the prevalence of preexisting comorbidities, which were identified using codes collected and reported within the dataset for each patient (Appendix, Table 6). Preexisting comorbidities included coronary artery disease, congestive heart failure, diabetes mellitus, cerebrovascular accident, peripheral vascular disease, pulmonary disease, chronic kidney disease (including stages 1–5 based on the National Kidney Foundation practice guidelines (Levey et al. 2003)), alcoholism, and a current smoking history. Hospital length of stay and ICU length of stay were summarized using calendar days.

We identified those who were diagnosed with hospital complications using codes collected and reported within the dataset for each patient (Appendix, Table 7). Hospital complications included acute kidney injury, acute respiratory distress syndrome (ARDS), cardiac arrests, cerebrovascular accidents, decubitus ulcer, deep vein thrombosis, alcohol or drug withdrawal, myocardial infarction, pneumonia, pulmonary embolism, unplanned intubation, urinary tract infection, and sepsis. The frequency and types of complications were reported for all patients, and for patients admitted to the ICU, complications were also reported by injury mechanism. Patients may have had more than one complication during the hospitalization. Bivariate analyses were conducted to assess the relationship between patient factors and the development of hospital complications using the *χ*^2^ and *t* test, as appropriate.

Multiple logistic regression analyses were performed to assess factors associated with the development of hospital complications and hospital mortality. Based on the results of Thompson *et al.* in creating the Mortality Risk for Trauma Comorbidity Index (Thompson et al. 2010) and the recommendation for rigorous risk-adjusted analysis of trauma mortality by Haider et al.(2012), the multivariable logistic models for hospital complications and hospital mortality included variables that assessed the mechanism of injury (e.g., blunt, penetrating), the physiologic severity (e.g., presence of hypotension on admission, head injury with severity **≥**4 on Abbreviated Injury Scale (AIS)), anatomic severity (e.g., Injury Severity Score (ISS)), age, and gender, as well as preexisting comorbidities.

Statistical anlysis was performed using Stata 12.1. We used the Stata survey procedures to account for the sampling design and sampling weights to account for differential probability of selection between strata. Weighted frequencies and proportions for each group were calculated on the basis of the relative weights for patients in each facility within the sample. Continuous variables are presented as medians with interquartile range (IQR). Missing data were rare and less than 5 % for all variables included in the analysis. The study was reviewed and approved by the New York Presbyterian-Columbia University Medical Center Institutional Review Board.

## Results

**Fig. 1:**
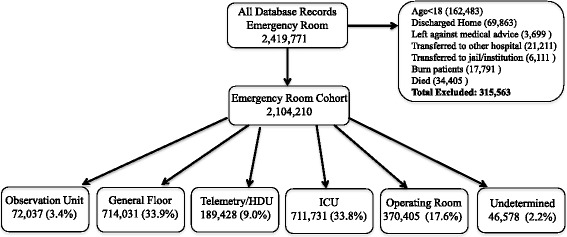
Patient flows from Emergency Room. *Numbers do not add up exactly due to rounding of weighted estimates. **Does not include all patients admitted to ICU, as temporal flow of admissions not included in dataset

The NTDB 2013 National Sample Program included 2,104,210 weighted records of hospital admission for traumatic injury at Level 1 and Level 2 trauma centers in 2013, and 1,028,817 (48.9 %) included ICU admission during the index hospitalization. The majority (61.3 %) of these patients were treated in ACS Level 1 trauma hospitals. Patients were admitted from the Emergency Room to various units in the hospital, including 711,731 (33.8 %) admitted directly to the ICU (Fig. 1). The national incidence of adult ICU admission after trauma was 3.3 per 1000 (95 % CI 3.2–3.3). The incidence was highest amongst those over 80 years of age (7.8 per 1000 (95 % CI, 7.7–8.0)) (Table 1).

**Table 1:** National Incidence of Intensive Care Unit Admissions after Traumatic Injury, National Trauma Data Bank, 2013

	Admissions to ICU after Trauma (95 % Confidence Interval)
Total	3.3 (3.2–3.3)
Gender	
Male	4.6 (4.6–4.6)
Female	1.9 (1.9–1.9)
Age, years	
18–39	4.1 (4.1–4.2)
40–59	3.6 (3.6–3.7)
60–79	4.3 (4.2–4.3)
≥80	7.8 (7.7-8.0)
Race^a^	
White	2.9 (2.9–2.9)
Black	3.1 (3.0–3.2)
Injury Class	
Blunt	2.9 (2.8–2.9)
Penetrating	0.3 (0.2–0.3)
Other^b^	0.1 (0.1–0.1)
Intention	
Assault	0.4 (0.4–0.4)
Self-Harm	0.1 (0.1–0.1)
Unintentional	2.8 (2.8–2.8)
US Census Region	
Northeast	2.7 (2.7–2.8)
South	2.9 (2.8–2.9)
Midwest	3.6 (3.6–3.7)
West	3.9 (3.8–3.9)

^a^Other races (e.g., Native Americans, Asian-Americans, Native Hawaiians) not stratified for analysis because precise stratified information on these sub-categories was not available in the dataset

^b^Other injuries includes environmental injuries (e.g., animal attack), drownings, overdoses or toxic ingestions, suffocation, exertional injuries, and unspecified injuries

Male patients made up 69.4 % of the ICU population, and the median age for all patients was 47 years (IQR 30-63). The majority (88.5 %) of patients were admitted to the ICU after blunt trauma, and the majority of injuries were unintentional (85.6 %). The most commonly injured body regions were the head (39.3 %) and extremities (25.0 %). The most common pre-existing comorbidities were alcoholism (13.2 %), current smoking status (17.3 %) and diabetes mellitus (10.8 %).

The development of hospital course complications was more common amongst patients admitted to the ICU than amongst patients hospitalized without ICU admission (22.6 versus 2.8 %, *p* < 0.001). Amongst patients admitted to the ICU the median number of complications was 1 (IQR 1–2, range 1–7). Patients who developed hospital complications were older, had more comorbidities, and more severe injuries than patients who did not develop complications (Table 2). The most common hospital complications amongst ICU patients were pneumonia (10.9 %), urinary tract infection (4.7 %), and ARDS (4.4 %) (Table 3). Factors which conferred the highest odds ratio for hospital complications in patients admitted to the ICU were mechanical ventilation (OR 6.7 (95 % CI, 6.4–7.1) *p* < 0.001), preexisting pulmonary disease (OR 4.7 (95 % CI, 2.6–8.4), *p* < 0.001), and Injury Severity Score **≥** 16 (OR 4.0 (95 % CI, 3.6–4.4), *p* < 0.001) (Table 4). Details on the distribution of hospital course complications stratified by mechanism of injury are available in the Additional file 1: Table S1.

**Table 2:** Descriptive statistics of hospitalized adults admitted to the ICU versus not admitted to the ICU after trauma including those with hospital course complications, National Trauma Data Bank, 2013^a^

	Admitted to ICU, weighted frequency (%)			Not admitted to ICU, weighted frequency (%)		
	Total	With Complications	*p*-value	Total	With Complications	*p*-value^b^
Total	1,028,817 (100)	232,618 (22.6)	<0.001	1,075,393 (100)	30,350 (2.8)	<0.001
Male	712,904 (69.4)	172,967 (74.5)	<0.001	681,396 (63.5)	20,934 (69.1)	<0.001
Age, years^a^	47 (30–63)	50 (33–63)	<0.001	47 (31–64)	56 (42–72)	<0.001
ACS Trauma Designation			<0.001			<0.001
Level 1	630,679 (61.3)	152,942 (65.7)		600,481 (55.8)	19,634 (64.7)	
Level 2	398,138 (38.7)	79,676 (34.3)		474,912 (44.2)	10,716 (35.3)	
Number of complications	NA	1 (1–2)		NA	1 (1–1)	<0.001
Injury Class			0.253			<0.001
Blunt	905,149 (88.5)	206,317 (89.1)		949,370 (88.3)	27,543 (90.8)	
Penetrating	89,461 (8.7)	18,693 (8.1)		87,510 (8.1)	1838 (6.1)	
Other^c^	28,239 (2.8)	6626 (2.9)		38,024 (3.5)	942 (3.1)	
Intention			<0.001			<0.001
Assault	115,665 (11.3)	22,026 (9.5)		139,440 (12.9)	2669 (8.8)	
Self-harm	23,860 (2.3)	5787 (2.5)		10.989 (1.0)	373 (1.2)	
Unintentional	875,604 (85.6)	201.794 (87.1)		919,374 (85.5)	27,191 (89.7)	
Undetermined	7720 (0.8)	2028 (0.9)		5102 (0.5)	90 (0.3)	
ISS^a^	17 (10–27)	26 (17–34)	<0.001	9 (5–12)	10 (5–17)	<0.001
GCS Score	15 (10–15)	13 (3–15)	<0.001	15 (15–15)	15 (14–15)	<0.001
Hypotension on Admission (SBP < 90 mmHg)	75,195 (7.4)	27,310 (12.1)	<0.001	18,049 (1.7)	2148 (7.1)	<0.001
Site of Injury^d^			<0.001			<0.001
Head	402,462 (39.3)	80,917 (34.9)		307,578 (28.7)	7575 (25.0)	
Thorax	150,598 (14.7)	38,725 (16.7)		114,816 (10.7)	3692 (12.2)	
Spine	118,202 (11.5)	30,944 (13.3)		92,583 (8.6)	2368 (7.8)	
Abdomen	75,112 (7.3)	20,155 (8.7)		41,600 (3.9)	2018 (6.7)	
Extremity	256,043 (25.0)	57,498 (24.8)		483,327 (45.1)	14,056 (46.4)	
Other	21,583 (2.1)	3598 (1.6)		31,794 (2.9)	582 (1.9)	
Comorbidities						
Alcoholism	135,774 (13.2)	41,254 (17.7)	<0.001	105,255 (9.8)	8066 (26.6)	<0.001
Cerebrovascular Accident	21,504 (2.1)	6718 (2.9)	<0.001	22,046 (2.1)	1989 (6.6)	0.603
Chronic Kidney Disease	9808 (1.0)	3877 (1.7)	<0.001	9878 (0.9)	1551 (5.1)	0.489
Congestive Heart Failure	29,195 (2.8)	8206 (3.5)	<0.001	32,259 (2.9)	1881 (3.5)	0.067
Coronary Artery Disease	15,056 (1.5)	4910 (2.1)	<0.001	15,327 (1.4)	2368 (7.8)	0.463
Current Smoker	177,724 (17.3)	40,623 (17.5)	0.425	207,069 (19.3)	7184 (23.7)	<0.001
Diabetes Mellitus	111,095 (10.8)	29,573 (12.7)	<0.001	116,138 (10.8)	5905 (19.5)	0.993
Peripheral Vascular Disease	2815 (0.3)	904 (0.4)	0.002	2925 (0.3)	344 (1.1)	0.944
Pulmonary Disease	1317 (0.1)	717 (0.3)	<0.001	1021 (0.1)	197 (0.6)	0.060
Mechanical ventilation	517,950 (50.3)	193,722 (83.3)	<0.001	30,641 (2.8)	2825 (9.3)	<0.001
Intensive Care Unit LOS^a^, days	4 (2–8)	12 (6–21)	<0.001	NA	NA	<0.001
Hospital LOS^a^, days	4 (2–8)	12 (6–21)	<0.001	3 (2–6)	7 (3–11)	<0.001
Hospital mortality	109,580 (10.7)	39,204 (16.9)	<0.001	15,716 (1.5)	4991 (16.4)	<0.001

*ISS* Injury Severity Score, *GCS* Glasgow Coma Scale, *ACS* American College of Surgery, *SBP* systolic blood pressure, *LOS* length of stay

^a^Continuous variables presented as sample median (Interquartile Range)

^b^ This value reflects analysis between those admitted to ICU who had hospital complications and those not admitted to ICU who had hospital complications

^c^Other injuries includes environmental injuries (e.g., animal attack), drownings, overdoses or toxic ingestions, suffocation, exertional injuries, and unspecified injuries

^d^Head includes Abbreviated Injury Scale scores for Head, Face, and Neck. Extremity includes all four extremities

**Table 3:** Hospitalized patients admitted to the ICU versus not admitted to the ICU after trauma, with hospital course complications by type ^a^, National Trauma Data Bank, 2013*

	Admitted to ICU, weighted frequency (%)	Not Admitted to ICU, weighted frequency (%)
Acute Kidney Injury	20,593 (2.0)	3192 (0.3)
ARDS	45,280 (4.4)	1570 (0.1)
Cardiac Arrest	21,145 (2.1)	4559 (0.4)
Cerebrovascular Accident	21,504 (2.1)	22,046 (2.1)
Decubitus Ulcer	23,160 (2.3)	1390 (0.1)
Deep Vein Thrombosis	43,283 (4.2)	4155 (0.4)
Drug/Alcohol Withdrawal	23,061 (2.2)	7291 (0.7)
Myocardial Infarction	5771 (0.6)	994 (0.1)
Pneumonia	112,221 (10.9)	6300 (0.6)
Pulmonary Embolism	12,759 (1.2)	1968 (0.2)
Unplanned Intubation	25,586 (2.5)	267 (0.0)
Urinary Tract Infection	48,695 (4.7)	10,587 (0.9)
Sepsis	15,476 (1.5)	478 (0.0)
Total	232,618 (22.6)	30,350 (2.8)

*ARDS* acute respiratory distress syndrome

*Differences between those admitted to ICU and those not admitted to ICU different in all categories, significant at *p* < 0.001

^a^Patients may have developed more than one complication

**Table 4:** Adjusted odds ratios, with 95 % Confidence Intervals (CI), of factors associated with hospital course complications and hospital mortality in trauma patients admitted to the ICU, National Trauma Data Bank, 2013

	Hospital Complications	*p*-value	Hospital Mortality	*p*-value
	OR (95 % CI)		OR (95 % CI)	
Gender				
Female	Reference		Reference	
Male	1.3 (1.2–1.3)	<0.001	1.4 (1.3–1.5)	<0.001
Age, years				
18–39	Reference		Reference	
40–59	1.4 (1.3–1.5)	<0.001	1.5 (1.4–1.6)	<0.001
60–79	1.8 (1.7–1.9)	<0.001	3.5 (3.2–3.8)	<0.001
>80	1.5 (1.4–1.7)	<0.001	15.9 (14.1–17.9)	<0.001
ISS category				
<9	Reference		Reference	
9–15	1.8 (1.6–1.9)	<0.001	2.2 (1.7–2.7)	<0.001
16+	4.0 (3.6–4.4)	<0.001	7.3 (5.9–8.9)	<0.001
Injury Class				
Blunt	Reference		Reference	
Penetrating	0.9 (0.8–1.1)	0.294	1.0 (0.9–1.2)	0.621
Other^a^	1.1 (0.9–1.2)	0.280	1.0 (0.9–1.2)	0.903
Intention				
Assault	Reference		Reference	
Self-harm	0.9 (0.7–1.0)	0.078	2.2 (1.8–2.6)	<0.001
Unintentional	1.1 (0.9–1.2)	0.218	0.9 (0.8–1.1)	0.299
GCS Score				
>12	Reference		Reference	
9–11	0.9 (0.8–0.9)	<0.001	3.1 (2.7–3.5)	<0.001
3–8	1.1 (1.1–1.2)	<0.001	6.6 (6.1–7.1)	<0.001
Hypotension on Admission (SBP < 90 mmHg)	1.2 (1.2–1.3)	<0.001	2.1 (1.9–2.3)	<0.001
Severe Head Injury^b^	0.8 (0.7–0.8)	<0.001	1.8 (1.7–1.9)	<0.001
Mechanical ventilation	6.7 (6.4–7.1)	<0.001	7.7 (7.0–8.5)	<0.001
Preexisting Comorbidities				
Alcoholism	1.8 (1.7–1.9)	<0.001	0.8 (0.8–0.9)	<0.001
Cerebrovascular Accident	1.6 (1.4–1.7)	<0.001	1.3 (1.1–1.5)	0.001
Chronic Kidney Disease	1.9 (1.5–2.4)	<0.001	2.0 (1.5–2.6)	<0.001
Congestive Heart Failure	1.7 (1.5–1.9)	<0.001	1.8 (1.5–2.1)	<0.001
Coronary Artery Disease	1.6 (1.4–1.8)	<0.001	1.0 (0.8–1.2)	0.882
Current Smoker	1.2 (1.1–1.2)	<0.001	0.5 (0.4–0.5)	<0.001
Diabetes Mellitus	1.3 (1.2–1.3)	<0.001	1.1 (0.9–1.2)	0.341
Peripheral Vascular Disease	1.4 (0.9–1.9)	0.076	4.4 (2.6–7.3)	<0.001
Pulmonary Disease	4.7 (2.6–8.4)	<0.001	0.3 (0.1–0.5)	<0.001
ACS Trauma Designation				
Level 2	Reference		Reference	
Level 1	1.1 (1.1–1.2)	<0.001	0.9 (0.8–0.9)	<0.001
Developed Hospital Complications (Yes/No)^c^	–	–	0.7 (0.6–0.8)	<0.001
Number of Complications (range 0–7)			2.3 (1.8–2.8)	<0.001
Acute Kidney Injury	–	–	0.9 (0.6–1.2)	0.376
ARDS	–	–	0.6 (0.5–0.7)	<0.001
Cardiac Arrest	–	–	9.5 (7.3–12.5)	<0.001
Cerebrovascular Accident	–	–	2.1 (1.5–2.9)	<0.001
Decubitus Ulcer	–	–	0.2 (0.2–0.3)	<0.001
Deep Vein Thrombosis	–	–	0.1 (0.1–0.2)	<0.001
Drug/Alcohol Withdrawal	–	–	0.2 (0.1–0.3)	<0.001
Myocardial Infarction	–	–	0.9 (0.6–1.3)	0.443
Pneumonia	–	–	0.2 (0.2–0.3)	<0.001
Pulmonary Embolism	–	–	0.3 (0.2–0.4)	<0.001
Unplanned Intubation	–	–	0.5 (0.4–0.6)	<0.001
Urinary Tract Infection	–	–	0.5 (0.4–0.5)	<0.001
Sepsis	–	–	2.3 (1.9–2.9)	<0.001

*CI* confidence interval, *ISS* Injury Severity Score, *GCS* Glasgow Coma Scale, *ACS* American College of Surgery, *SBP* systolic blood pressure, *ARDS* acute respiratory distress syndrome

^a^Other injuries includes environmental injuries (e.g., animal attack), drownings, overdoses or toxic ingestions, suffocation, exertional injuries, and unspecified injuries

^b^Severe Head injury includes Abbreviated Injury Scale scores for Head, Face, and Neck with severity greater than or equal to 4

^c^Reference is no complications

Hospital mortality for patients admitted to the ICU who had hospital course complications was significantly higher than those admitted to the ICU who did not develop hospital course complications (16.9 % versus 10.7 %, *p* < 0.001). Factors which increased the odds of hospital mortality in patients admitted to the ICU included age over 80 years (OR 15.9 (95 % CI, 14.1–17.9), *p* < 0.001), mechanical ventilation (OR 7.7 (95 % CI, 7.0–8.5), *p* < 0.001), Injury Severity Score **≥**16 (OR 7.3 (95 % CI, 5.9–8.9), *p* < 0.001), Glasgow Coma Scale Score between 3 and 8 (OR 6.6 (95 % CI, 6.1–7.1), *p* < 0.001), and hospital complications including in-hospital cardiac arrest (OR 9.5 (95 % CI, 7.3–12.5), *p* < 0.001). Each hospital complication increased the odds ratio for hospital mortality by 2.3 ((95 % CI, 1.8–2.8) *p* < 0.001) (Table 4).

## Discussion

This study describes the characteristics and outcomes for adult patients admitted to the ICU at Level 1 and Level 2 trauma hospitals after traumatic injury in the United States. We found that almost half of patients hospitalized after trauma were admitted to the ICU. This population was primarily composed of young males with blunt unintentional traumatic injuries. Hospital complications developed in almost a quarter (22.6 %) of patients who required ICU admission and were associated with a higher severity of injury (median ISS 26 (IQR 17–34)). Although hospital mortality for patients admitted to the ICU (10.7 %) was not high compared to other ICU cohorts in the US, hospital mortality amongst those with hospital course complications (16.9 %) was significantly higher and similar to that of ICU populations nationwide (Lilly et al. 2011).

There is scant epidemiological literature describing patients who require critical care services after trauma in the United States. Epidemiological investigations of trauma patients admitted to the ICU have been conducted in other high-income countries (Curtis et al. 2012), developing countries (Chalya et al. 2011; Adenekan 2009) and in military settings (Brown et al. 2011), but these studies lack generalizability to the United States because of variations in the availability and structure of pre-hospital systems and different ICU bed availability. Although descriptive studies have been conducted in the United States, these studies are often single-center (Ong et al. 2009) or focused on very specific subsets of patients (Brown et al. 2011; Majidi et al. 2014; Nishijima et al. 2013; Lustenberger et al. 2011; Recinos et al. 2009; Sangthong et al. 2006), which also limits generalizability. One recent multicenter study described the case mix, complications, and outcomes of 11,064 patients admitted to ICUs after trauma, and found that hospital complications were associated with age, gender, and traumatic CNS injury (Mondello et al. 2014). The rigor of our study is attributable to the large number of patients from hospitals across the country, the prospective validated data collection process, and the representative nature of the data.

Although ICU admission for traumatic injury, at least for some period of observation, is common practice in many centers (Kaufman et al. 2016), regional triage criteria and the actual utilization of ICU-level care (e.g., mechanical ventilation) are unclear. The severity of illness for patients admitted to the ICU in this study was only moderately high (median ISS 17, IQR 10–27). ICU admission for observation-only may theoretically result in unnecessarily high healthcare costs (Wunsch et al. 2008), exposure of patients to ICU-related complications (e.g., nosocomial infections (Grundmann et al. 2005) and medical errors during transfers of care (Bell et al. 2011)), and denial of ICU beds to other patients. Similarly, ICU admission for patients with extremely high expected mortality may be considered futile, and may also result in high healthcare costs and delayed ICU admission for other patients (Huynh et al. 2014). Admission decisions are often subjective, and for these reasons it is important to clarify the optimal use of ICU resources. This study may serve as a first step to informing ICU triage decisions for trauma patients. For example, these data demonstrate a higher severity of injury amongst patients with hospital course complications who were admitted to the ICU (median ISS 26, IQR 17–34). Although we cannot establish whether hospital course complications occurred before or after ICU admission, hospital course complications may be one clinical factor utilized to determine whether some proportion of patients may be safely treated in other hospital areas, such as intermediate care units, without adversely affecting outcomes.

Additionally, these data demonstrate that 50.3 % of trauma patients admitted to the ICU receive mechanical ventilation, while up to 9.3 % of trauma patients admitted to other hospital areas also require mechanical ventilation. With the exception of patients intubated for airway protection (i.e., central nervous system trauma, airway hemorrhage, penetrating chest trauma), alternatives to mechanical ventilation may be explored for some patients to reduce the prevalence of associated complications and mortality. For example, non-invasive pressure support ventilation has demonstrated a mortality benefit in adult trauma patients (Roberts et al. 2014; Chiumello et al. 2013). The reasons behind the relatively high provision of mechanical ventilation in non-ICU settings deserves further attention.

The hospital complication rate amongst patients admitted to the ICU was high (22.6 %). With increasing scrutiny on quality of care and recent linking of complication rates to reimbursement (Sipkoff 2008), healthcare systems nationwide are searching for ways to reduce in-hospital complications. An important target of future research should include clarifying the time course of hospital complications in hospitalized trauma patients, so as to better identify modifiable risk factors. Preexisting comorbidities were also common in the ICU cohort, especially alcoholism, smoking, and diabetes, despite a generally young population. Notably, these three common comorbidities were not strong predictors of hospital complications and were not associated with an increased risk of hospital mortality. Although the mechanism underlying this association is beyond the scope of this study, these findings are consistent with the results of a single-center study evaluating smoking and trauma outcomes (Ferro et al. 2010). The comorbidities most strongly associated with hospital complications and mortality (pulmonary disease, peripheral vascular disease, and chronic kidney disease) were rare in the cohort. This type of information may help guide triage decisions and future study design.

An important limitation of these data is the lack of temporal association between ICU admission and the development of hospital complications. We do not suggest that hospital complications lead to ICU admission, or vice versa. This important data would greatly enhance the ability to draw clinical conclusions from this study. This study also includes only those patients admitted to American College of Surgery Level 1 and Level 2 designated trauma centers. Despite well-developed trauma triage systems in the United States, some proportion of patients will be treated at Level III or IV centers, or non-trauma centers, and this study does not include these patients. Another limitation is the lack of detailed clinical data about intra-hospital patient transfers, staffing, rapid response teams, and ICU organization. Finally, although the sampling population of hospitals in the National Sample Program is intended to be nationally representative, this likely increases the heterogeneity of ICU and hospital organization within the cohort. Organizational hospital-level factors have been shown to affect outcomes (Sakr et al. 2015) and these limitations limit the generalizability and interpretation of these results.

## Conclusions

ICU admission after traumatic injury in adults is common, and almost a quarter of these patients will develop hospital course complications. Hospital complications are associated with significantly higher hospital mortality for ICU patients, and more detailed clinical data is necessary to identify modifiable risk factors. Describing the characteristics and hospital course of patients admitted to the ICU after traumatic injury is an important first step to clarifying the needs of this population.

## Abbreviations

ACS, American College of Surgery; AIS, Abbreviated Injury Scale; ARDS, acute respiratory distress syndrome; CI, confidence intervals; GCS, Glasgow Coma Scale; ICU, intensive care unit; IQR, interquartile range; ISS, Injury Severity Score; LOS, length of stay; NTDB, National Trauma Data Bank; SBP, systolic blood pressure

## Appendix

**Table 5:** United States 2013 national population, stratified by gender, age-group, race, and census region (United States Census Bureau 2013)^a^

United States population, total	316,128,839
Gender	
Male	155,651,602
Female	160,477,237
Age, years	
18–39	95,307,000
40–59	84,983,000
60–79	49,910,000
>80	10,988,000
Race	
White	245,499,216
Black	41,623,897
Other^b^	21,264,998
Census Region	
Northeast	55,943,073
Midwest	67,547,890
South	118,383,453
West	74,254,423

^a^United States 2013 population data as of July 1, 2013, accessed through https://www.census.gov/population/age/data/2013comp.html on May 9, 2016

^b^Other includes Native Americans, Asian-Americans, Native Hawaiians

**Table 6:** National Trauma Data Bank National Sample Program, 2013 data codes of pre-existing comorbidities utilized in analysis

Pre-existing comorbidities	Source in dataset
Coronary Artery Disease^a^	NTDB comorbidity codes 16, 17 ICD9 codes 414.01, 440.9, 414.00, 414.4, 414.3, 414.02
Congestive Heart Failure	NTDB comorbidity code 7
Diabetes Mellitus	NTDB comorbidity code 11
Cerebrovascular Accident	NTDB comorbidity code 10
Peripheral Vascular Disease	NTDB comorbidity code 18
Pulmonary Disease	ICD9 codes 490.0, 490, 491.0, 491.1, 491.2, 491.20, 491.22, 491.8, 491.9, 492.0, 492.8, 493, 493.0, 493.1, 493.2, 494.0, 493.1, 495.0, 495.1, 495.2, 495.3, 495.4, 495.5, 495.6, 495.7, 495.8, 495.9, 496.0
Chronic Kidney Disease	ICD9 codes 585.1, 585.2, 585.3, 585.4, 585.5, 585.6, 585.9
Alcoholism	NTDB comorbidity code 2
Current Smoker	NTDB comorbidity code 8

^a^Because the NTDB dataset used multiple comorbidity codes to indicate coronary artery disease (e.g., history of a myocardial infarction and history of angina), additional ICD9 codes were queried to ensure no cases were missing

**Table 7:** National Trauma Data Bank National Sample Program, 2013 data codes of hospital complications utilized in analysis

Complications	Source in dataset
Acute Kidney Injury	NTDB complication code 4 ICD9 584.5-584.9, 588.0-588.9, 585.89, 585.9, 593.9, 958.5
ARDS	NTDB complication code 5 ICD9 518.5, 518.82
Cardiac Arrest	NTDB complication code 8 ICD9 427.5 in conjunction with 99.60-99.69, 427.5 with 37.91; v12.53
Cerebrovascular Accident	NTDB complication code 22 ICD9 434.01, 434.11, 434.91, 433.01-433.91, 997.02
Decubitus Ulcer	NTDB complication code 11 707.00 through 707.09
Deep Vein Thrombosis	NTDB complication code 14 ICD9 451.0, 451.11, 451.19, 451.2, 451.81-451.84, 451.89, 451.9, 453.40, 459.10-459.19, 997.2, 999.2
Alcohol/Drug Withdrawal	NTDB complication code 13 ICD9 291.0, 291.3, 291.81, 292.0
Myocardial Infarction	NTDB complication code 18 IC9 414.8, 412
Pneumonia	NTDB complication code 20 IC9 480.0-480.9, 481, 482.0-482.3, 482.30-483.39, 482.40-482.49, 482.81-482.89, 482.9, 483.0-483.8, 484.1-484.8, 485, 486, 997.31
Pulmonary Embolism	NTDB complication code 21 ICD9 415.11, 415.12, 415.19, 416.2
Unplanned intubation	NTDB complication code 25 “Patient requires placement of an endotracheal tube and mechanical or assisted ventilation because of the onset of respiratory or cardiac failure manifested by severe respiratory distres, hypoxia, hypercarbia, or respiratory acidosis. In patients who were intubated in the field or Emergency Department, or those intubated for surgery, unplanned intubation occurs if they require reintubation >24 h after extubation.” *NTDB User Manual 2013*
Urinary Tract Infection	NTDB complication code 27 ICD9 595.0-595.9 or 599.0
Sepsis	NTDB complication code 32 ICD9 785.52, 995.92

## Additional file

Additional file 1:
**Table S1.** Weighted frequency and percentage of patients admitted to the ICU with hospital course complications by type, stratified by mechanism of injury, National Trauma Data Bank, 2013. (DOC 43 kb)
