# Supplementary material for: Complications and in-hospital mortality in trauma patients treated in intensive care units in the United States, 2013
Source: Inj Epidemiol. 2016 Aug 4;3(1):18. doi: 10.1186/s40621-016-0084-5 (PMC4974260; doi:10.1186/s40621-016-0084-5)
Supplement: Additional file 1: — Table S1. Weighted frequency and percentage of patients admitted to the ICU with hospital course complications by type, stratified by mechanism of injury, National Trauma Data Bank, 2013. (DOC 43 kb) [file 40621_2016_84_MOESM1_ESM.doc]

Additional file 1: Table S1. Weighted frequency and percentage of patients admitted to the ICU with hospital course complications by type, stratified by mechanism of injury, National Trauma Data Bank, 2013^a^

|  | Blunt Trauma | Penetrating Trauma | Other Trauma^b^ | p-value |
| --- | --- | --- | --- | --- |
|  | Weighted Frequency (%) | Weighted Frequency (%) | Weighted Frequency (%) |  |
| Acute Kidney Injury | 17,861 (1.9) | 1,646 (1.8) | 636 (2.3) | 0.641 |
| ARDS | 40,429 (4.5) | 3,542 (3.9) | 1,303 (4.6) | 0.465 |
| Cardiac Arrest | 18,208 (2.0) | 2,634 (2.9) | 233 (0.8) | 0.189 |
| Cerebrovascular Accident | 10,654 (1.1) | 1,391 (1.6) | 300 (1.1) | 0.366 |
| Decubitus Ulcer | 21,493 (2.4) | 1,061 (1.2) | 489 (1.7) | <0.001 |
| Deep Vein Thrombosis | 38,928 (4.3) | 3,521 (3.9) | 722 (2.6) | <0.001 |
| Drug/Alcohol Withdrawal | 21,176 (2.3) | 800 (0.9) | 947 (3.4) | 0.326 |
| Myocardial Infarction | 5,411 (0.6) | 326 (0.4) | 34 (0.1) | <0.001 |
| Pneumonia | 100,330 (11.1) | 7,852 (8.8) | 3,750 (13.3) | 0.947 |
| Pulmonary Embolism | 11,534 (1.3) | 1,004 (1.1) | 197 (0.7) | <0.001 |
| Unplanned intubation | 23,630 (2.6) | 1,480 (1.7) | 476 (1.7) | <0.001 |
| Urinary Tract Infection | 44,594 (4.9) | 2,546 (2.8) | 1,554 (5.5) | 0.003 |
| Sepsis | 14,177 (1.6) | 894 (0.9) | 308 (1.1) | <0.001 |
| Total | 206,317 (22.8) | 18,693 (20.9) | 6,626 (23.5) | 0.254 |

^a^Patients may have had more than one complication

^b^Other injuries includes environental injuries (e.g. animal attack), drownings, overdoses or toxic ingestions, suffocation, exertional injuries, and unspecified injuries

Acute respiratory distress syndrome (ARDS)
